# Low confidence for perceptual completion of partially occluded objects

**DOI:** 10.1167/jov.26.1.4

**Published:** 2026-01-06

**Authors:** Cemre Baykan, Pascal Mamassian, Alexander C. Schütz

**Affiliations:** 1 Philipps-Universität Marburg, Fachbereich Psychologie, AG Sensomotorisches Lernen, Germany; 2 Laboratoire des Systèmes Perceptifs, Département d’Études Cognitives, École Normale Supérieure, PSL University, CNRS, Paris, France

**Keywords:** perceptual completion, amodal completion, modal completion, visual confidence, metacognition

## Abstract

Pervasive gaps in sensory information are completed in perception. Interestingly, humans are unaware of that perceptual completion in cases of proximal gaps, which are caused by properties of their own sensory system, and report high confidence for the inferred information in those gaps. Here, we investigated whether such overconfidence is also observed in perceptual completion of visual information in distal gaps (i.e., those caused by the properties of the stimulus). In three experiments, we asked participants to perform a perceptual (type 1) task and report their confidence (type 2 task) using stimuli that were either intact (full stimulus), with a partial cutout (stimulus with gap), partially occluded (amodal completion) or induced (modal completion). We examined whether participants report high confidence for amodal and modal completion in comparison to a full stimulus or stimulus with gap. Over three experiments, participants had the highest confidence for full stimuli, whereas amodal and modal completion led to comparable confidence as stimuli with gap. These findings demonstrate that there was low confidence for stimuli whose distal gaps are perceptually filled in. In combination with previous research, our results suggest that visibility of the gaps in information influences confidence judgments.

## Introduction

Our visual system lacks sensory information due to various reasons. Anatomically, in the blind spot regions, where the optic nerve leaves the eye and where there are no photoreceptors, no light can be detected (Durgin, Tripathy, & Levi, 1995; Ramachandran, 1992). In the fovea, where there are no rod photoreceptors, no light can be detected under low-light conditions (Curcio, Sloan, Kalina, & Hendrickson, 1990; Jacobs, 2002). Angioscotomata, where blood vessels cast shadows on the photoreceptors in the retina, also lead to a loss of information (Adams & Horton, 2002; Mark, 2014; Purkinje, 1825). Apart from anatomical reasons, there are environmental factors that constrict the available visual information such as occlusion of an object by other objects or by its own parts (Nanay, 2018). Despite these factors, we have a seamless perceptual experience of visual scenes by means of “filling-in” mechanisms whereby the brain substitutes the missing information (Michotte, Thinès, & Crabbé, 1967; Pessoa, Thompson, & Noë, 1998; Ramachandran & Gregory, 1991). Earlier studies showed that some forms of perceptual completion or filling-in occur outside of our awareness, leading to overconfidence for filled-in stimuli in the blind-spot (Ehinger, Häusser, Ossandón, & König, 2017; Ramachandran, 1992), in the foveal rod-scotoma (Gloriani & Schütz, 2019), and also in the auditory sense (Baykan, Mamassian, & Schütz, 2025). Yet, it is not known whether observers have similar metacognitive distortions for filled-in (inferred) visual stimuli when some of their parts are physically absent.

Metacognition is often described as one's subjective assessment of their own cognitive state and can be measured with confidence judgments (Fleming, Dolan, & Frith, 2012; Lau & Rosenthal, 2011; Mamassian, 2016). Typically, metacognitive ability is assessed as observers make a perceptual decision, referred to as type 1 task, and a confidence judgment about that response, referred to as type 2 task (Galvin, Podd, Drga, & Whitmore, 2003; Maniscalco & Lau, 2012). Previous research demonstrated that humans have good metacognitive abilities: their confidence judgments correlate with the actual decision performance (Barthelmé & Mamassian, 2009; Desender, Boldt, Verguts, & Donner, 2019; Fleming & Dolan, 2012). However, there have been some examples showing that perceptual performance and confidence judgments do not align when individuals perform a perceptual task involving filled-in stimuli (Baykan et al., 2025; Ehinger et al., 2017; Gloriani & Schütz, 2019). For example, Ehinger et al. (2017) examined the visual completion in proximal gaps (i.e., those caused by our own sensory systems) and found that observers selected the filled-in stimulus presented inside the blind spot as more continuous compared to a physically identical stimulus presented outside the blind spot. Similarly, we previously explored auditory completion of information in distal gaps (i.e., those caused by the properties of the stimulus). We found that listeners were unaware of auditory filling-in and trusted a filled-in auditory stimulus as much as a veridical one (Baykan et al., 2025). Here, we tested whether observers are overconfident for filled-in stimuli caused by perceptual completion in distal gaps in vision.

There are some cases in which visual stimuli physically lack some of its parts and yet are perceived as complete (Gerbino, 2020; Pessoa & De Weerd, 2003; Scherzer & Faul, 2019; Weil & Rees, 2011). Amodal completion is a process in which objects are perceived as complete even though they are partially occluded (Michotte et al., 1967; Thinés, Costall, & Butterworth, 2013), whereas modal completion is a mechanism in which observers perceive illusory contours or borders when parts of an object are obscured by matching color and brightness with its background (Kanizsa, 1979; Tse, 1999). Earlier studies have suggested that these two types of completion share several common characteristics (Durgin et al., 1995; Gerbino, 2020; Ramachandran, 1995; Wagemans, 2015). Both amodal and modal completion occur when there is no direct sensory stimulation (Kellman & Shipley, 1991), and are encoded at early stages of visual processing (Sereno & Maunsell, 1998). Moreover, both phenomena can be driven by early visual (bottom-up) and higher order (top-down) processes (Thielen, Bosch, van Leeuwen, van Gerven, & van Lier, 2019). Early visual processes include the use of stimulus features such as contour alignment, continuation or T-junctions (Albert & Hoffman, 2000; Kellman & Shipley, 1991; Watanabe & Cavanagh, 1993), whereas higher-order processes rely on familiarity or prior knowledge (Hazenberg & van Lier, 2016). Despite these similarities, amodal and modal completion exhibit differences in neural correlates (Lee, 2003; Sugita, 1999), spatial distribution of visual attention (i.e., the spread of attention between the filled-in regions and their inducers [Davis & Driver, 1997]) and perceptual accuracy [Spehar & Halim, 2016]. Both phenomena involve the brain's ability to fill in gaps, but they exhibit differences in neural responses and mechanisms.

In the present study, we examined perceptual performance for stimuli with and without completion and the confidence for these decisions. We ran three separate experiments, in which three groups of participants performed orientation discrimination, Vernier acuity, and continuity-discontinuity decision tasks. In each experiment, there were four stimulus conditions: full stimulus, stimulus with gap, amodal completion and modal completion. We predicted that if individuals trust filled-in stimuli caused by perceptual completion in distal gaps in vision, similar to other cases of perceptual completion (Baykan et al., 2025; Ehinger et al., 2017; Gloriani & Schütz, 2019), they should report comparable or even higher confidence for amodally and modally completed stimuli relative to full stimulus. However, if they are aware of filling-in and do not trust filled-in stimuli, their confidence judgments should be lower than the ones for the full stimulus.

## Methods

### Participants

Data were collected from 71, 51 and 78 participants online (on the platform prolific.com) with normal or corrected-to-normal vision in experiments 1 to 3, respectively. Supplementary Table S1 summarizes the exclusion criteria for all experiments. In Experiment 1 (preregistration https://aspredicted.org/sntx-msvd.pdf), we excluded participants with less than 75% valid trials. A trial was considered invalid if the response time was ≤ 300 ms or ≥ 5000 ms, assuming that response times below 300 ms are anticipatory responses that are not stimulus-driven and response times above 5000 ms are cases in which participants were distracted by something else. We also calculated a regression of confidence on performance and excluded participants if the regression slopes were <0.25 or >5 (indicating that the metacognitive sensitivity was poor and that confidence ratings were not related to the actual stimulus and performance). In Experiment 2 (preregistration https://aspredicted.org/k7q6-rzjt.pdf), we excluded participants with less than 80% valid trials. The definition of a valid trial was identical to Experiment 1. We also excluded participants if the proportion of correct responses to the smallest or largest offset condition was less than 70%. Compared to the preregistration, we dropped the criterion that each confidence level had to be used at least five times, because too many participants would have been excluded (19 additional participants). In Experiment 3 (preregistration https://aspredicted.org/2bjv-b62q.pdf), the valid datasets were determined based on simultaneous fulfillment of the following criteria: for the non-ambiguous full stimulus condition, the proportion of the correct responses was >0.85 (indicating that participants were performing the task accurately and did not produce too many errors) and preference of this stimulus on the confidence judgment was >0.68 (indicating that participants preferred the non-ambiguous stimulus over the uncertain stimuli). We lowered that value from 0.85 in the preregistration to 0.68 because with 32 trials, 22 versus 10 choices is the smallest imbalance that reaches significance in an uncorrected McNemar test. Furthermore, with 0.85 an unreasonably large proportion (40%) of participants would have needed to be excluded. The number of participants that were excluded based on these criteria are shown in Supplementary Table S1. We also reanalyzed the whole datasets of Experiments 1 and 2 without excluding any participant, which resulted in similar findings (see Supplementary Material).

As a result, we entered the data of 46 participants (15 females, 29 males, one non-binary, one unreported; age range, 19–43 years; mean age = 27.6 years across 45 participants) to the main analysis in Experiment 1, 37 participants (23 females, 14 males; age range, 18–43 years; mean age = 25.7 years of 36 participants) in Experiment 2, and 55 participants (26 females, 29 males; age range 19–44 years; mean age of 26.6 years) in Experiment 3. All participants were naive to the purpose of the study and gave written informed consent before the experiments started. Each participant was compensated by a monetary payment of 7 pounds per hour in Experiment 1, and 8 pounds per hour in Experiments 2 and 3, for their participation. The experiments were approved by the local ethics committee of the Psychology Department at the University of Marburg (proposal number 2020-43k).

### Stimuli and Procedure

**Figure 1.**
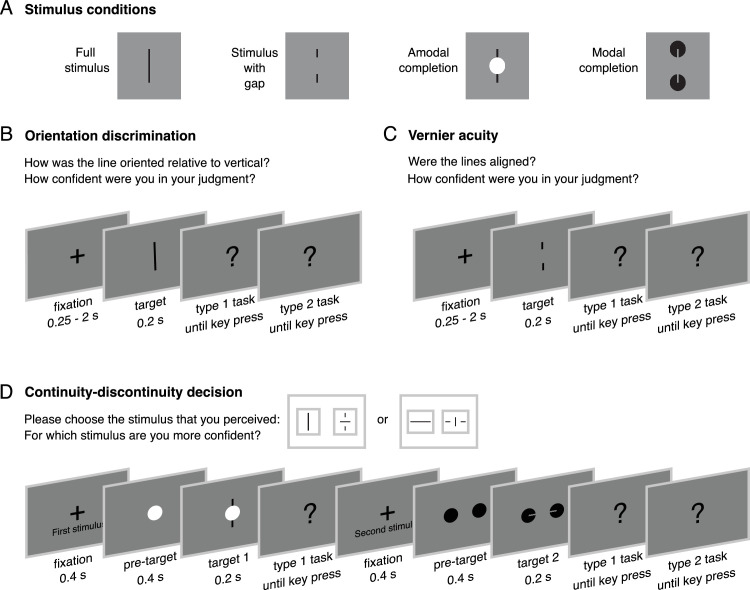
Illustration of a trial procedure. (**A**) In three experiments, there were four stimulus conditions: full stimulus, stimulus with gap, amodal completion and modal completion (see Stimuli and Procedure for details). (**B**) In Experiment 1, participants performed an orientation discrimination task. Each trial started with a fixation cross for a randomly chosen interval between 0.25 to two seconds. After this, an oriented line as target stimulus was shown for 0.2 second. Next, participants made a perceptual decision (type 1 task) indicating how the line was oriented relative to the vertical. Finally, they made a confidence judgment (type 2 task) reporting how confident they were for their decision on that stimulus. (**C**) In Experiment 2, participants performed a Vernier acuity task. Each trial started with a fixation cross for a randomly chosen interval between 0.25 to 2 seconds. After this, two equally-long adjacent line segments either with or without offsets as target stimulus were shown for 0.2 second. Next, participants made a perceptual decision (type 1 task) indicating if these lines were aligned. Finally, they made a confidence judgment (type 2 task) reporting how confident they were for their decision on that stimulus. (**D**) In Experiment 3, participants performed a continuity task for two consecutive stimuli. Each trial started with a fixation cross for 0.4 second. After this, a pre-target period for 0.4 second took place in which blank screen was shown for full stimulus and stimulus with gap conditions, whereas discs were shown for amodal and modal completion conditions. Then, a line as target stimulus was shown for 0.2 second. Next, participants made a perceptual decision (type 1 task) indicating how their perception of that stimulus was. They had two response options: a line with its center aligned (continuous stimulus) and with its center rotated by 90° (discontinuous stimulus). The response options could be vertical or horizontal depending on the target stimulus. After participants’ response, a second stimulus was shown following the same sequence. Finally, participants made a confidence judgment (type 2 task) reporting for which of their perceptual reports they were more confident.

The experiments were programmed and presented using jsPsych (de Leeuw, 2015). All stimuli were presented on a gray (R = G = B = 128) background. In each experiment, there were four types of stimuli: “full stimulus” consisting of a complete shape, “stimulus with gap” consisting of the same shape with a partial cutout, “amodal completion” stimulus having the same shape with its cutout placed behind an occluder, and “modal completion” stimulus that has two inducers to generate the full stimulus shape (Figure 1A). The stimulus was a black line (length 64 pixels, width 4 pixels) in the full stimulus condition. In the stimulus with gap condition, the center of the line was interrupted by a gap (size: half of the length of the line), whereas it was covered by a white disc in the amodal completion condition. In the modal completion condition, the line was of the background color and appeared on top of two aligned black discs (distance between the centers and disc diameter equal to the half of the length of the line).

In Experiment 1, participants performed an orientation discrimination task (Figure 1B). Each trial started with a black fixation cross (60 pixels) that was centrally presented for a random duration chosen from the set [250, 500, 750, 1000, 1250, 1500, 1750, 2000] ms. Next, the stimulus was presented for 200 ms. Stimuli were oriented lines with tilts ranging from −2° to 2° with a step of 0.25°. Afterward, a response screen appeared where participants were asked to select whether the stimulus line was rotated counterclockwise or clockwise relative to the vertical. Participants made their orientation decisions by selecting one of the two options (clockwise or counterclockwise tilted lines) presented on screen. Then, participants made a confidence judgment about their perceptual decision using a four-point scale (“Not at all,” “Slightly,” “Quite,” “Very”). After each part of the trial (fixation cross, stimulus presentation, response screen, confidence judgment), a 200-ms blank screen was shown. Participants completed 320 trials: 10 repetitions for four stimulus conditions and eight orientations.

In Experiment 2, participants performed a Vernier acuity task (Figure 1C). Each trial started with a black fixation cross (60 pixels) that was centrally presented for a random duration chosen from the set (250, 500, 750, 1000, 1250, 1500, 1750, 2000) ms. Next, the stimulus was presented for 200 ms. Stimuli were two equally-long adjacent line segments oriented either vertically or horizontally. The segments had offsets (vertical for horizontal segments, horizontal for vertical segments) selected from the set (−8; −4; −3; −2; 0; 2; 3; 4; 8) pixels. After the stimulus, a response screen appeared where participants were asked to indicate if the line segments were aligned. Participants made their choices by selecting one of the “yes” or “no” options. Then, participants made a confidence judgment about their perceptual decision using the four-point scale. After each part of the trial (fixation cross, stimulus presentation, response screen, confidence judgment), a 200-ms blank screen was shown. Participants completed 360 trials: 10 repetitions for four stimulus conditions and nine offsets.

In Experiment 3, participants performed a continuity-discontinuity decision task (Figure 1D), similar to the studies on the blind spot and the foveal rod scotoma (Ehinger et al., 2017; Gloriani & Schütz, 2019). In this experiment, participants made two consecutive continuity decisions and a two-alternative forced choice confidence judgment. Each trial started with a black fixation cross (60 pixels) that was centrally presented and accompanied by a text “First stimulus”. This presentation lasted until a button press and was followed by a blank screen for 400 ms. Before the first stimulus, there was a “pre-target” part for another 400 ms, in which a blank screen was kept for the full stimulus and stimulus with gap conditions, whereas discs (without target lines) were shown for amodal and modal completion conditions. This was done to strengthen the completion perception and emphasize the lines as distinct entities separate from occluders. Next, the stimulus was presented for 200 ms, followed by a blank screen for another 200 ms. Stimuli were horizontal or vertical black lines, and either continuous or discontinuous (if discontinuous, the central segment was rotated by 90 degrees relative to the outer sections). In the discontinuous stimuli, the rotation of the central segment was clearly visible for the full stimulus condition, whereas this rotation was invisible in the stimuli with gap, amodal and modal completion given that their central segment was either cutout, occluded or induced, respectively. After the first stimulus, a response screen appeared where participants were asked to indicate which stimulus they perceived among two options (center of the line aligned or orthogonal to its outer segments). Following their response, the same sequence including fixation cross (with an accompanying text “Second stimulus”), pre-target, target and response presentations took place for the second stimulus. Then, participants made a confidence forced-choice judgment, reporting for which perceptual decision out of two (first or second) they were more confident by clicking a respective button. Participants completed 192 trials: 12 repetitions for four stimulus conditions, two continuous or discontinuous (orthogonal), and two vertical or horizontal. In the full stimulus condition, we used two types of stimuli: continuous or discontinuous, whereas in the other three stimulus conditions, there was only a single type of stimulus. The order of stimuli was counterbalanced to avoid any influence of interval biases for the first or second stimulus.

### Statistical analyses

All data analyses were performed in R (version 4.3.2; R Core Team, 2023). In Experiment 1 and 2, we calculated standardized confidence values using z-score transformation by taking the difference between the confidence ratings and the mean and dividing these values by the standard deviation, per participant. This was done because we were not interested in the absolute level of confidence and because we wanted to eliminate individual differences in the usage of confidence scales (Dienes, 2016; Pallier et al., 2010). We analyzed the perceptual decisions and confidence ratings using a linear mixed-effects model via *lme4* (version 1.1–35.1, Bates, Mächler, Bolker, & Walker, 2015) package, and compared the group means in linear models using estimated marginal means via *emmeans* (version 1.10.0, Lenth, 2024) package. The preregistrations contained a simpler analysis of variance (ANOVA), which was ultimately not used because line orientation or offset could not be treated as continuous variable. In Experiment 3, we analyzed the perceptual decisions and confidence judgments using one-way repeated-measure ANOVA via *ez* (version 4.4.0, Lawrence, 2016) package and pairwise *t*-test via *stats* (version 4.3.2, R Core Team 2023) package. In all three experiments, we also reported response consistency of perceptual decisions, that is the stability of making the same decision for a given stimulus (Caziot & Mamassian, 2021). This was done to examine whether participants estimated how consistent they were when they were prompted to make confidence judgments on the accuracy of their perceptual decisions (see Caziot & Mamassian, 2021; Koriat, 2025). In Experiment 1, we calculated response consistency in each trial, by multiplying the stimulus orientation and response values, with counterclockwise and clockwise stimuli and responses being coded as −1 and 1, respectively. Responses for counterclockwise stimuli were inverted. In Experiments 2 and 3, we calculated response consistency by calculating the absolute distance of proportion of perceptual responses to the subjective equality between continuous and discontinuous (i.e., 0.5) and multiplying the result by 2. In Experiment 3, we used the same metric. In the full stimulus condition, response consistency was calculated separately for continuous and discontinuous stimuli (because they were clearly discriminable), whereas they were combined in the other conditions (because they were indiscriminable).

## Results

### Experiment 1: Orientation discrimination

**Figure 2.**
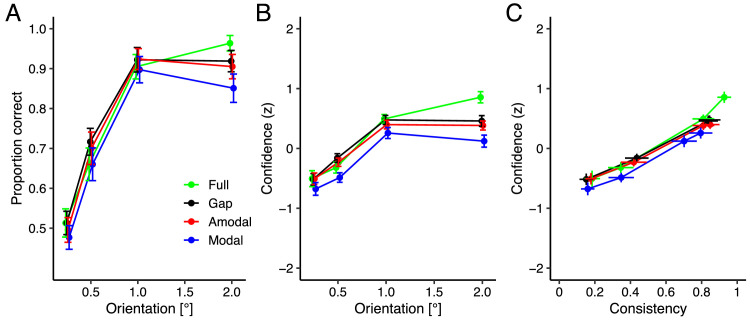
Results of Experiment 1: Orientation discrimination. (**A**) Proportion of correct responses and (**B**) Confidence ratings (*z*-scores) as a function of line orientation. (**C**) Confidence ratings (*z*-scores) as a function of response consistency. Green represents the full stimulus; black the stimulus with gap; red the amodal completion; blue the modal completion conditions. Error bars depict 95% confidence intervals.

In Experiment 1, we first asked participants to perform a line orientation discrimination task where they had to decide whether a line stimulus was tilted clockwise or counterclockwise relative to the vertical and then to rate their confidence using a four-point scale rating. Figure 2A shows proportion correct as a function of line orientation. We examined the discrimination performance of different stimulus conditions by employing a linear mixed-effects model on the proportion correct with stimulus condition as a categorical predictor (full stimulus coded as a dummy variable) and line orientation as a continuous predictor, including a random intercept and slope of the predictors by participant. There was a significant intercept (*b* = 0.56, *SE* = 0.01, *t* = 50.44, *p* < 0.001). The expected proportion correct for stimulus with gap (*b* = 0.01, SE = 0.01, *t* = 0.73, *p* = 0.47) and amodal completion (*b* = −0.003, *SE* = 0.01, *t* = −0.30, *p* = 0.76) were comparable to full stimulus, while the expected proportion correct for modal completion was significantly lower than full stimulus, *b* = −0.04, *SE* = 0.01, *t* = −3.78, *p* < 0.001. Furthermore, there was a significant slope of line orientation across all stimulus conditions, *b* = 0.21, *SE* = 0.01, *t* = 30.79, *p* < 0.001. We performed pairwise comparisons between stimulus conditions by comparing estimated marginal means with Tukey adjustment. The expected proportion correct for the stimulus with gap was significantly higher compared to modal completion (difference = 0.05, *t* = 4.71, *p* < 0.001), whereas it was comparable to amodal completion (difference = 0.01, *t* = 1.06, *p* = 0.72). Moreover, the expected proportion correct for amodal completion was significantly higher compared to modal completion (difference = 0.04, *t* = 3.64, *p* = 0.004). These results demonstrated that participants performed the orientation discrimination comparably for the conditions of full stimulus, stimulus with gap and amodal completion, with significantly poorer performance for the condition of modal completion.

Figures 2B and 2C show confidence as a function of line orientation and response consistency, respectively. The mean (± standard error [*SE*]) confidence was for the full stimulus 0.13 (±0.02), for the stimulus with gap 0.06 (±0.02), for amodal completion 0.01 (±0.02) and for modal completion −0.20 (±0.03). We examined the confidence judgments of different stimulus conditions, line orientations and response consistencies by using a linear mixed-effects model on the confidence judgments with stimulus condition as a categorical predictor (full stimulus coded as a dummy variable) and line orientation and response consistency as continuous predictors, including a random intercept and slope of the predictors by participant. There was a significant intercept (*b* = −0.44, *SE* = 0.05, *t* = −9.43, *p* < 0.001). The expected confidence for the stimulus with gap (*b* = −0.07, *SE* = 0.03, *t* = −2.48, *p* = 0.02), amodal completion (*b* = −0.12, *SE* = 0.03, *t* = −3.52, *p* < 0.001) and modal completion (*b* = −0.31, *SE* = 0.04, *t* = −7.31, *p* < 0.001) were significantly lower than for the full stimulus. Furthermore, there was a significant slope of line orientation (*b* = 0.46, *SE* = 0.03, *t* = 13.99, *p* < 0.001) and response consistency (*b* = 0.25, *SE* = 0.01, *t* = 18.24, *p* < 0.001) across all stimulus conditions. Pairwise comparisons using Tukey's honestly significant difference test revealed that the expected confidence for the stimulus with gap was significantly higher compared to modal completion (difference = 0.24, *t* = 6.87, *p* < 0.001), whereas it was comparable to amodal completion (difference = 0.05, *t* = 2.03, *p* = 0.19). Moreover, the expected confidence for amodal completion was significantly higher compared to modal completion (difference = 0.19, *t* = 6.11, *p* < 0.001). Hence, the full stimulus condition resulted in the highest confidence, the amodal and gap conditions demonstrated a lower confidence, and modal completion had the lowest confidence rating.

Experiment 1 used an orientation discrimination task for line stimuli with or without gap. It might be that the presence or absence of a gap (and therefore also perceptual completion) was immaterial for the orientation discrimination task, such that perceptual completion did not affect the metacognitive confidence judgments. Therefore, in Experiment 2, we used interrupted or discontinuous line stimuli where the two segments of them had small Vernier offsets. Perceptual completion should have a closer relationship with the type 1 task in this case.

### Experiment 2: Vernier acuity

**Figure 3.**
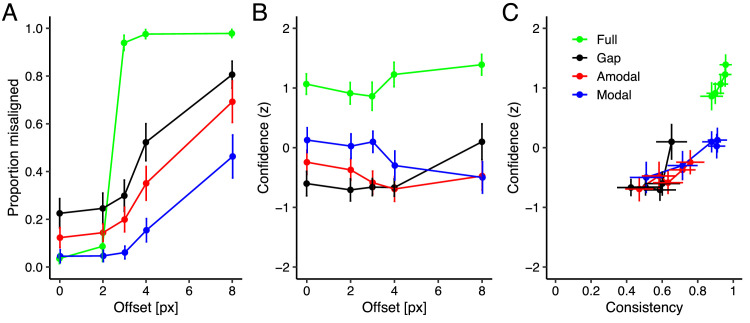
Results of Experiment 2: Vernier acuity. (**A**) Proportion of misaligned decisions as a function of lines offset in pixels. (**B**) Confidence ratings (*z*-scores) as a function of lines offset. (**C**) Confidence ratings (*z*-scores) as a function of response consistency. Green represents the full stimulus; black the stimulus with gap; red the amodal completion; blue the modal completion conditions. Error bars depict 95% confidence intervals.

In Experiment 2, we asked participants to make a perceptual decision about whether two line segments were aligned or not, and to rate their confidence using a four-point scale rating. Figure 3A illustrates the proportion of misaligned responses as a function of line offset. The mean (±*SE*) proportion misaligned was for the full stimulus 0.67 (± .01), for the stimulus with gap 0.44 (±0.03), for amodal completion 0.32 (±0.02) and for modal completion 0.17 (±0.02). We examined the alignment responses of different stimulus conditions by using a linear mixed-effects model on the proportion misaligned with stimulus condition as a categorical predictor (full stimulus coded as a dummy variable) and line offset as a continuous predictor, including a random intercept and slope of the predictors by participant. There was a significant intercept (*b* = 0.34, *SE* = 0.02, *t* = 19.05, *p* < 0.001). The expected proportion misaligned for the stimulus with gap (*b* = −0.22, *SE* = 0.02, *t* = −9.40, *p* < 0.001), amodal completion (*b* = −0.35, *SE* = 0.02, *t* = −14.85, *p* < 0.001) and modal completion (*b* = −0.50, *SE* = 0.02, *t* = −30.87, *p* < 0.001) were significantly lower than for the full stimulus. Furthermore, there was a significant slope of line offset across all stimulus conditions, *b* = 0.09, *SE* = 0.004, *t* = 22.18, *p* < 0.001. We performed pairwise comparisons between stimulus conditions by comparing estimated marginal means with Tukey adjustment. The expected proportion misaligned for the stimulus with gap was significantly higher compared to amodal completion (difference = 0.12, *t* = 6.69, *p* < 0.001) and modal completion (difference = 0.28, *t* = 11.65, *p* < 0.001), and the expected proportion misaligned for amodal completion was significantly higher compared to modal completion (difference = 0.15, *t* = 7.36, *p* < 0.001). To summarize, the misalignments were best detected in the full stimulus condition, worse in the stimulus with gap condition, even worse in the amodal completion and the worst in the modal completion condition. This is evidence for perceptual completion in the modal and amodal condition, which biases participants to report the stimuli as aligned.

Figures 3B and 3C show the confidence as a function of line offset and response consistency, respectively. The mean (±*SE*) confidence was for the full stimulus 1.09 (±0.06), for the stimulus with gap −0.51 (±0.05), for amodal completion −0.47 (±0.05) and for modal completion −0.11 (±0.07). We examined the confidence judgments of different stimulus conditions, line offsets and response consistencies by using a linear mixed-effects model on the confidence judgments with stimulus condition as a categorical predictor (full stimulus coded as a dummy variable) and line offset and response consistency as continuous predictors, including a random intercept and slope of the predictors by participant. There was a significant intercept (*b* = −0.34, *SE* = 0.13, *t* = −2.66, *p* = 0.01). The expected confidence for the stimulus with gap (*b* = −1.10, *SE* = 0.09, *t* = −12.52, *p* < 0.001), for amodal completion (*b* = −1.17, *SE* = 0.09, *t* = −13.48, *p* < 0.001) and for modal completion (*b* = −1.02, *SE* = 0.10, *t* = −9.68, *p* < 0.001) were significantly lower than full stimulus. Furthermore, there was a significant slope of confidence judgments as a function of line offset (*b* = 0.04, *SE* = 0.01, *t* = 3.61, *p* < 0.001), and response consistency (*b* = 1.41, *SE* = 0.10, *t* = 13.63, *p* < 0.001), across all stimulus conditions. Post-hoc comparisons using Tukey's honestly significant difference test revealed that the expected confidence for the stimulus with gap was comparable to amodal completion (difference = 0.07, *t* = 1.21, *p* = 0.63) and modal completion (difference = −0.09, *t* = −0.91, *p* = 0.80), and the expected confidence for amodal completion was comparable to modal completion (difference = −0.16, *t* = −1.89, *p* = 0.25). These results showed that confidence rating was the highest for the full stimulus condition.

Previous studies, reporting overconfidence for inferred information in sensory gaps (Baykan et al., 2025; Ehinger et al., 2017; Gloriani & Schütz, 2019) used tasks, in which observers had to discriminate between continuous and discontinuous stimuli, which is a trivially easy task outside of the perceptual scotoma. In contrast, the Vernier task in this experiment was challenging even without gaps or perceptual completion. Hence, we used a simple continuity-discontinuity task in the next experiment. Confidence ratings provide direct assessments of one's confidence, yet bear several disadvantages (e.g., being sensitive to individual differences in generation and use of ratings internally and having the confounding effect of the stimulus strength [visibility] in confidence response) (Aitchison, Bang, Bahrami, & Latham, 2015; Mamassian, 2016; Rausch & Zehetleitner, 2016). Accordingly, in Experiment 3, we used a confidence forced-choice paradigm.

### Experiment 3: Continuity-discontinuity decisions

**Figure 4.**
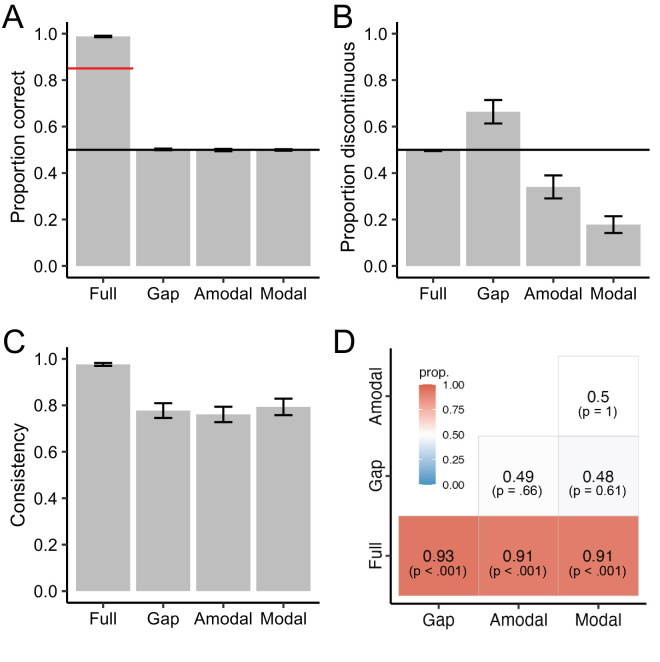
Results of Experiment 3: Continuity-discontinuity decisions. (**A**) Proportion of correct responses, (**B**) proportion of discontinuous responses and (**C**) response consistency across stimulus conditions. (**D**) Participants’ interval preference for reporting their perceptual decision as more “confident” for the conditions on the y-axis relative to the conditions on the x-axis. Color bar and large numbers represent the proportion of participants’ preference. The *p* values are indicated in parentheses. Horizontal black lines show the chance level (**A**, **B**). Error bars depict 95% confidence intervals (**A**–**C**).

In Experiment 3, participants performed a continuity-discontinuity decision (as in previous studies on the blind spot and the foveal rod scotoma; Ehinger et al., 2017; Gloriani & Schütz, 2019) for two consecutive stimuli and indicated their confidence using the confidence forced-choice paradigm, selecting the decision that they felt more confident about. Figures 4A–C show proportion correct, proportion discontinuous and response consistency for different stimulus conditions, respectively. We ran three separate one-way repeated-measures ANOVAs to examine the effect of the stimulus condition. First, we analyzed proportion correct as a measure of how accurate participants discriminated the stimuli. The mean (± standard error, *SE*) proportion correct was at ceiling for the full stimulus 0.99 (±0.003), and at chance for all the other conditions, for the stimulus with gap 0.50 (±0.003), for amodal completion 0.50 (±0.01) and for modal completion 0.50 (±0.003).

In the second step, we analyzed the proportion of discontinuous decisions as a measure of perceptual biases. Continuous and discontinuous stimuli were presented in 50% of the trials each. If there is perceptual completion in the modal and amodal stimulus, the proportion of discontinuous decisions should be less than 50%. The mean (±*SE*) proportion discontinuous was for the full stimulus 0.50 (±0.002), for the stimulus with gap 0.66 (±0.05), for amodal completion 0.34 (± 0.05) and for modal completion 0.18 (±0.04). The results showed that there was a main effect of stimulus condition on proportion discontinuous, *F*(3, 216) = 27.51, *p* < 0.001, $\eta_{g}^{2}$ = 0.28. The pairwise *t*-test results revealed that the proportion discontinuous for the full stimulus significantly differed from any other condition: stimulus with gap, *t*(54) = −3.30, *p* = 0.002, *d* = 0.63; amodal completion, *t*(54) = 3.18, *p* = 0.003, *d* = 0.60; and modal completion, *t*(54) = 8.85, *p* < 0.001, *d* = 1.68. Moreover, the proportion discontinuous for the stimulus with gap significantly differed from amodal completion, *t*(54) = 5.50, *p* < 0.001, *d* = 0.87, and modal completion, *t*(54) = 8.36, *p* < 0.001, *d* = 1.49. The proportion discontinuous for amodal completion also significantly differed from modal completion, *t*(54) = 3.07, *p* = 0.003, *d* = 0.50. The finding that observers responded with more “continuous” responses suggests that there was perceptual completion for amodal and modal completion, with stronger effects on modal completion.

Finally, we analyzed the response consistency. The mean (±*SE*) response consistency was for the full stimulus 0.98 (±0.01), for the stimulus with gap 0.78 (±0.03), for amodal completion 0.76 (±0.03) and for modal completion 0.79 (±0.04). The results showed that there was a main effect of stimulus condition on response consistency, *F*(3, 216) = 11.87, *p* < 0.001, $\eta_{g}^{2}$ = 0.14. The pairwise *t*-test results revealed that response consistency for the full stimulus significantly differed from any other condition: stimulus with gap, *t*(54) = 6.45, *p* < 0.001, *d* = 1.17; amodal completion, *t*(54) = 6.52, *p* < 0.001, *d* = 1.22; and modal completion, *t*(54) = 5.26, *p* < 0.001, *d* = 0.97. None of the other comparisons were significant, *t*s(54) < 0.53, *p*s > 0.48, *d*s < 0.13.

Figure 4D illustrates participants’ interval preference for reporting their perceptual decision as more “confident” across different stimulus conditions. A one-way repeated measures ANOVA showed that there was a significant main effect of stimulus condition on stimulus preference, *F*(5, 324) = 75.14, *p* < 0.001, $\eta_{g}^{2}$ = 0.54. First, we analyzed whether the preference for the full stimulus over other stimulus conditions differed from 50%. A one-sample *t*-test against 0.5 indicated that the preference for the full stimulus was significantly higher than 0.5, 95% CI = [0.90, 0.93], *t*(164) = 59.95, *p* < 0.001, and there was no significant difference among the preference for the full stimulus over any other stimulus condition, *F*(2, 162) = 0.54, *p* = 0.59, $\eta_{g}^{2}$ = 0.01. This result indicated that the confidence judgments are in agreement with response consistency. Next, we compared the preference for a given condition against 0.5 chance level using separate one-sample *t*-tests. The results revealed that there was no preference for a stimulus with gap over amodal (95% confidence interval [CI] = [0.42, 0.55], *t*(54) = −0.45, *p* = 0.66) or modal completion (95% CI = [0.41, 0.56], *t*(54) = 0.52, *p* = 0.61), as well as no preference for an amodal stimulus over modal completion (95% CI = [0.42, 0.57], *t*(54) = 0, *p* > 0.99).

## Discussion

In this study, we aimed at investigating confidence for visual stimuli with perceptual completion. To this end, we compared the confidence judgments for full (intact) stimuli and stimuli with gap (a partial cutout) to amodally and modally completed stimuli. In the Vernier acuity task (Experiment 2) and the continuity-discontinuity task (Experiment 3), we observed that perceptual performance was lower for stimuli with gap, amodal completion and modal completion compared to full stimuli. In line with this, observers reported lower confidence for these three conditions relative to the full stimulus. Across three experiments with different tasks, we found that participants had the highest confidence for the full stimulus condition.

In the first experiment, using an orientation discrimination task, perceptual performance did not strongly vary between stimulus conditions, yet observers reported higher confidence for the full stimulus, indicating an increased trust for the full stimulus. In the second experiment, we manipulated Vernier offsets of line stimuli to increase the relevance of completion for the perceptual decision. In this experiment, stimulus conditions had a significant effect on perceptual responses: Vernier offset detection decreased from the full stimulus to the stimulus with gap, to amodal completion and further down to modal completion. Despite this, there was no large difference in confidence judgments between the conditions except for the full stimulus. This result indicates that participants had a comparably reduced confidence for stimuli with gap, amodal and modal completion. We also observed similar findings in the third experiment, showing that observers selected the full stimulus frequently as “confident” while having no preference among the other three conditions.

The main objective of this study was to investigate whether metacognitive judgments for stimuli whose distal gaps are perceptually filled-in would show similar biases to those in other cases of completion. We found that confidence for filled-in stimuli was lower than for full stimuli and comparable to stimuli with a gap. This observation is particularly interesting because it does not seem to align with previous findings that humans are overconfident in their inferences about occluded areas or for perceptually filled-in object information (Baykan et al., 2025; Ehinger et al., 2017; Gloriani & Schütz, 2019; Men, Altin, & Schütz, 2023).

One possible explanation of these disparate results could be that the type of perceptual completion plays a crucial role for perceptual decisions and decision confidence. Perceptual completion phenomena can be broadly grouped into two categories. The first involves filling-in of sensory information in proximal gaps that occurs due to anatomical reasons despite the presence of physical stimulation (Barton & Brewer, 2015; Durgin et al., 1995). The second engages perceptual filling-in of distal gaps that occurs in the absence of physical stimulation from the environment (Murray, Foxe, Javitt, & Foxe, 2004; Thinés et al., 2013). In the visual modality, Ehinger et al. (2017) and Gloriani and Schütz (2019) examined the decision confidence for filled-in perception that originates from completion at the blind spot and the foveal rod scotoma, respectively. In the case of perceptual filling-in at proximal gaps, it is likely that observers are not aware of missing sensory information or any occlusion. Consequently, they do not take the low reliability of inferred information into account while making confidence judgments. Unlike these studies, we tested visual filling-in at distal gaps in objects and found that decision confidence in stimuli with a gap, amodal and modal completion was similarly low in comparison to a full stimulus. This finding implies that seeing a physical gap or an occlusion seems to have similar effects on confidence judgments. Moreover, it supports the notion that visibility of missing sensory information or occlusion decreases confidence judgments.

A lack of awareness for missing sensory information or occlusion does not only occur for filling-in at proximal gaps. There have also been examples of filling-in at distal gaps showing that people were unaware of what might be missing, yet reported high confidence for filled-in stimuli (Baykan et al., 2025; Men et al., 2023). For instance, in the auditory modality, we previously found that listeners have comparable confidence for filled-in and veridical sounds when the completion phenomenon originates from masking by noise: participants judged continuous and discontinuous tones as similar when a masking noise was overlaid (Baykan et al., 2025). This implies that they were not aware of the masking of sensory information by the noise. Similarly, Men et al. (2023) examined participants’ numerosity estimation of visible and hidden objects in partially occluded scenes and asked participants to judge their decision confidence. Their results showed that observers underestimated the number of hidden objects behind the occlusion, but were similarly confident for their decisions in scenes with and without occlusion. This indicates that observers did not take into account for their confidence judgment that they are objectively missing information about objects that might be hidden behind the occlusion. In the current study, we used stimuli with partial cutout or stimuli that are partially occluded or induced. Although observers were aware of the partially missing information of occluded objects in this study, they apparently were not aware of the possible presence of completely occluded objects in partially occluded scenes (Men et al. 2023). These results point out that decision confidence is determined by awareness of occlusions and missing information, rather than the type of perceptual completion.

Our results suggest that awareness, that is conscious perception of the missing information, influences confidence. This result is in line with earlier studies which predict a close connection between confidence and awareness: A correlation between confidence and accuracy predicts stimulus awareness (Kunimoto, Miller, & Pashler, 2001; Persaud, McLeod, & Cowey, 2007). Accordingly, confidence judgments have been seen as a measure of conscious awareness (Dienes, 2004; Lau & Rosenthal, 2011). Importantly, however, several studies have reported that there are cases in which metacognition diverges from conscious processes, hence metacognitive judgments do not necessitate awareness of stimulus (Sahraie, Weiskrantz, Trevethan, Cruce, & Murray, 2002; Lau & Passingham 2006; but see Balsdon & Azzopardi, 2015). Specifically, research on neuropsychological conditions such as achromatopsia or blindsight provides strong evidence on this dissociation (Carota & Calabrese, 2013; Persaud et al., 2007; Persaud et al., 2011). For example, Persaud et al. (2011) examined confidence judgments of a patient with hemianopia, having no conscious visual experience in their impaired or blind visual field, a condition called blindsight. They found that the patient was confident of their responses in the blind visual field as much as in the intact field given that they wagered the same amount of money for both visual fields. Although this condition, that is having no awareness of stimulus in the blind field and yet reporting high confidence, is typically observed for blindsight type 2 (Sahraie, Weiskrantz, & Barbur, 1998; Weiskrantz, Barbur, & Sahraie, 1995), it is also reported for healthy observers (Zehetleitner & Rausch, 2013). There are several factors being proposed to decrease the correspondence between confidence judgments and perceptual decisions (for reviews, see Rahnev et al., 2022; Shekhar & Rahnev, 2021). Some of these factors have been shown to influence confidence whereas others only affect accuracy (e.g., confidence on previous trials and motor preparation affect confidence judgments) (Fleming et al., 2015; Mueller & Weidemann, 2008), while evidence congruent with decision predicts the response accuracy (Zylberberg, Barttfeld, & Sigman, 2012). Our study extends these findings and proposes that conscious awareness of missing information in perceptual completion is one of the factors that contributes to confidence computation.

One could expect that participants would be more aware of the missing information in stimuli with a gap than in stimuli with modal and amodal completion and therefore be less confident for the stimuli with a gap than with modal and amodal completion. We did not find any evidence for such a difference, indicating that although the participants experienced perceptual completion in the stimuli with modal and amodal completion, they were still aware that these percepts are not veridical.

In summary, we observed that participants have low confidence for modal and amodal filled-in stimuli compared to non-occluded stimuli. This observation does not align with earlier findings that found high confidence reports for inferred information in other cases of missing sensory information (Baykan et al., 2025; Ehinger et al., 2017; Gloriani & Schütz, 2019; Men et al., 2023). Together with these previous findings, our results suggest that confidence judgments for stimuli with perceptual completion is influenced by humans’ awareness of the gaps in information.

## Supplementary Material

Supplement 1
