# Supplementary material for: Low confidence for perceptual completion of partially occluded objects
Source: J Vis. 2026 Jan 6;26(1):4. doi: 10.1167/jov.26.1.4 (PMC12786398; doi:10.1167/jov.26.1.4)
Supplement: Supplement 1 [file jovi-26-1-4_s001.pdf]

Supplementary material for

Low confidence for perceptual completion of partially occluded objects

Cemre Baykan[1], Pascal Mamassian[2], Alexander C. Schütz[1]

[1] Philipps-Universität Marburg, Fachbereich Psychologie, AG Sensomotorisches Lernen, Germany

[2] Laboratoire des Systèmes Perceptifs, Département d'Études Cognitives, École Normale Supérieure, PSL University, CNRS, Paris, France.

Exclusion criteria

Supplementary Table 1: Summary of the exclusion criteria for all experiments.

Experiment	Exclusion criterion	Number of participants
1	More than 25% invalid trials (response time ≤300 ms or ≥5000 ms)	11
	Regression slope of confidence on performance <0.25 or >5	11
	Both	3
2	More than 20% invalid trials (response time ≤300 ms or ≥5000 ms)	8
	Proportion of correct responses in the smallest or largest offset condition less than 70%	3
	Both	3
3	Proportion of correct responses in the full stimulus less than 85%	2
	Preference of confidence judgment for full stimulus less than 68%	14
	Both	7

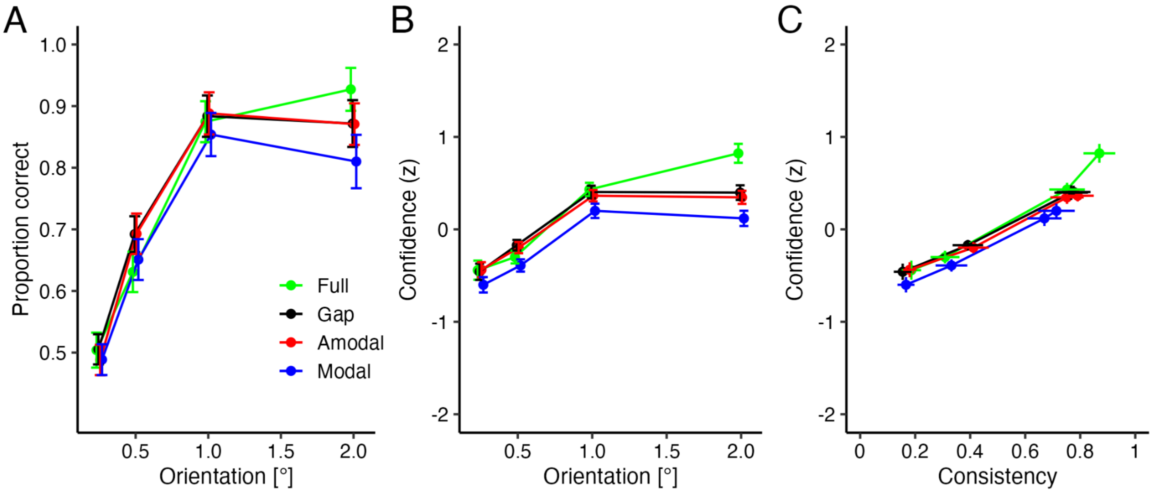

Figure S1: Results of Experiment 1 using the data of all 71 participants. Conventions are the same as in Figure 2.

Experiment 1

We reran the analysis including the data of all participants in Experiment 1 (Fig. S1). In general, the results are consistent with the analysis in the main text. Fig. S1A shows proportion correct as a function of line orientation in Experiment 1. We examined the discrimination performance of different stimulus conditions by employing a linear mixed-effects model on the proportion correct with stimulus condition as a categorical predictor (full stimulus coded as a dummy variable) and line orientation as a continuous predictor while participant was entered as random intercept. There was a significant intercept (b = 0.55, SE = 0.01, t = 46.84, p < .001). The expected proportion correct for stimulus with gap (b = 0.01, SE = 0.01, t = 0.73, p = .46) and amodal completion (b = 0.002, SE = 0.01, t = 0.26, p = .79) were comparable to full stimulus, while the expected proportion correct for modal completion was significantly lower than full stimulus, b = -0.03, SE = 0.01, t = -3.66, p < .001. Furthermore, there was a significant slope of line orientation across all stimulus conditions, b = 0.20, SE = 0.01, t = 44.34, p < .001. We performed pairwise comparisons between stimulus conditions by comparing estimated marginal means with Tukey adjustment. The expected proportion correct for the stimulus with gap was significantly higher compared to modal completion (difference = 0.04, t = 4.40, p < .001), while it was comparable to amodal completion (difference = 0.004, t = 0.47, p = .96). Moreover, the expected proportion correct for amodal completion was significantly higher compared to modal completion (difference = 0.03, t = 3.92, p = .001).

Fig. S1B and C show confidence as a function of line orientation and response consistency, respectively, in Experiment 1. We examined the confidence judgments of

different stimulus conditions, line orientations and response consistencies by employing a linear mixed-effects model on the confidence judgments with stimulus condition as a categorical predictor (full stimulus coded as a dummy variable) and line orientation and response consistency as continuous predictors while participant was entered as random intercept. There was a significant intercept (b = -0.39, SE = 0.02, t = -24.53, p < .001). The expected confidence for the stimulus with gap (b = -0.08, SE = 0.02, t = -4.20, p < .001), amodal completion (b = -0.11, SE = 0.02, t = -6.02, p < .001) and modal completion (b = -0.28, SE = 0.02, t = -15.28, p < .001) were significantly lower than for the full stimulus. Furthermore, there was a significant slope of line orientation (b = 0.42, SE = 0.01, t = 42.09, p < .001) and response consistency (b = 0.23, SE = 0.01, t = 30.62, p < .001) across all stimulus conditions. Pairwise comparisons using Tukey's HSD test revealed that the expected confidence for the stimulus with gap was significantly higher compared to modal completion (difference = 0.20, t = 11.09, p < .001), while it was comparable to amodal completion (difference = 0.03, t = 1.82, p = .26). Moreover, the expected confidence for amodal completion was significantly higher compared to modal completion (difference = 0.17, t = 9.27, p < .001).

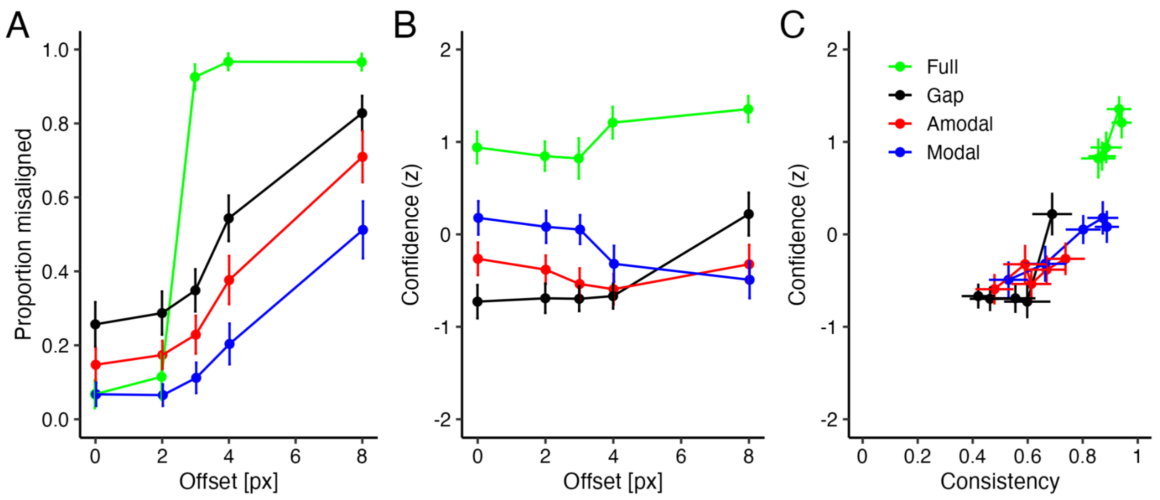

Figure S2: Results of Experiment 2 using the data of all 51 participants. Conventions are the same as in Figure 3.

Experiment 2

We reran the analysis including the data of all participants in Experiment 2 (Fig. S2). In general, the results are consistent with the analysis in the main text. Fig. S2A illustrates the proportion of misaligned responses as a function of line offset in Experiment 2. We examined the alignment responses of different stimulus conditions by employing a linear mixed-effects model on the proportion misaligned with stimulus condition as a categorical predictor (full stimulus coded as a dummy variable) and line offset as a continuous predictor while participant was entered as random intercept. There was a significant intercept (b = 0.35, SE = 0.02, t = 21.10, p < .001). The expected proportion misaligned for the stimulus with gap (b = -0.19, SE = 0.01, t = -22.30, p < .001), amodal completion (b = -0.32, SE = 0.01, t = -36.01, p < .001) and modal completion (b = -0.47, SE = 0.01, t = -52.47, p < .001) were significantly lower than for the full stimulus. Furthermore, there was a significant slope of line offset across all stimulus conditions, b = 0.09, SE = 0.001, t = 67.62, p < .001. We performed pairwise comparisons between stimulus conditions by comparing estimated marginal means with Tukey adjustment. The expected proportion misaligned for the stimulus with gap was significantly higher compared to amodal completion (difference = 0.12, t = 13.83, p < .001) and modal completion (difference = 0.27, t = 30.35, p < .001), and the expected proportion misaligned for amodal completion was significantly higher compared to modal completion (difference = 0.15, t = 16.48, p < .001).

Fig. S2B and C show the confidence as a function of line offset and response consistency, respectively, in Experiment 2. We examined the confidence judgments of different stimulus conditions, line offsets and response consistencies by employing a linear

mixed-effects model on the confidence judgments with stimulus condition as a categorical predictor (full stimulus coded as a dummy variable) and line offset and response consistency as continuous predictors while participant was entered as random intercept. There was a significant intercept (b = -0.35, SE = 0.08, t = -4.30, p < .001). This indicates that the expected confidence for the full stimulus was -0.35 when the line offset and response consistency were zero. The expected confidence for the stimulus with gap (b = -1.06, SE = 0.06, t = -18.50, p < .001), for amodal completion (b = -1.08, SE = 0.06, t = -19.73, p < .001) and for modal completion (b = -0.93, SE = 0.05, t = -17.91, p < .001) were significantly lower than full stimulus. Furthermore, there was a significant slope of confidence judgments as a function of line offset (b = 0.04, SE = 0.01, t = 5.66, p < .001), and response consistency (b = 1.40, SE = 0.07, t = 19.27, p < .001), across all stimulus conditions. Post-hoc comparisons using Tukey's HSD test revealed that the expected confidence for the stimulus with gap was comparable to amodal completion (difference = 0.03, t = 0.49, p = .96) and modal completion (difference = -0.12, t = -2.35, p = .09), and the expected confidence for amodal completion was lower than modal completion (difference = -0.15, t = -2.89, p = .02).
